# Horizontal Gene Transfer, Dispersal and Haloarchaeal Speciation

**DOI:** 10.3390/life5021405

**Published:** 2015-05-19

**Authors:** R. Thane Papke, Paulina Corral, Nikhil Ram-Mohan, Rafael R. de la Haba, Cristina Sánchez-Porro, Andrea Makkay, Antonio Ventosa

**Affiliations:** 1 Department of Molecular and Cell Biology, University of Connecticut, Storrs, CT 06269, USA; E-Mails: nikhil.ram_mohan@uconn.edu (N.R.-M.); andrea.makkay@uconn.edu (A.M.); 2 Department of Microbiology and Parasitology, University of Seville, 41004 Seville, Spain; E-Mails: pcv@us.es (P.C.); rrh@us.es (R.R.H.); sanpor@us.es (C.S.-P.); ventosa@us.es (A.V.)

**Keywords:** Haloarchaea, speciation, biogeography, homologous recombination, horizontal gene transfer, population genetics

## Abstract

The Halobacteria are a well-studied archaeal class and numerous investigations are showing how their diversity is distributed amongst genomes and geographic locations. Evidence indicates that recombination between species continuously facilitates the arrival of new genes, and within species, it is frequent enough to spread acquired genes amongst all individuals in the population. To create permanent independent diversity and generate new species, barriers to recombination are probably required. The data support an interpretation that rates of evolution (e.g., horizontal gene transfer and mutation) are faster at creating geographically localized variation than dispersal and invasion are at homogenizing genetic differences between locations. Therefore, we suggest that recurrent episodes of dispersal followed by variable periods of endemism break the homogenizing forces of intrapopulation recombination and that this process might be the principal stimulus leading to divergence and speciation in Halobacteria.

## 1. Background

In our search to understand the biological universe, a deep appreciation of diversity is required. This is, however, somewhat of an ambiguous declaration as diversity is multifaceted and occurs at many levels: alleles at a locus, genes in genomes, individuals in a population, populations comprising species, species in a community, *etc.* Though excellent arguments for the gene, or the individual as being the most basic unit in evolution [1,2], not until we examine and compare genetic diversity within populations or species are expansive patterns measured and revealed. Thus, species attracts our attention when contemplating the meaning of diversity. There is, however, a debate on how to define the term species, or which species concept is best [2,3,4], or even if they exist at all [5,6]. Nonetheless, species can be highly relevant for conceptualizing the stability of an ecosystem to perturbations, or for determining the impact of humans on the environment, or understanding the structure and function of any two communities. Further, it seems less complicated to identify when two individuals belong to different species—especially if they are distantly related—than it is to know when they belong to the same species. Unambiguously circumscribing species is a difficult undertaking: evolution is a never-ending process that generates incipient species inside incipient species. Therefore, we are tasked with attempting an ever better understanding of the speciation process in order to better recognize species.

Critical to understanding the process of speciation is recognizing how gene diversity is distributed amongst individuals so that the forces of evolution shaping that diversity can be elucidated. An important question to ask is: what are the evolutionary forces that produces individuals more similar in appearance to each other than to any other organisms—for instance, which evolutionary processes generate the observed clusters in phylogenetic trees? A second valuable question is: which processes promote the independent accumulation of variation and the formation of incipient lineages—*i.e.*, generate multiple closely related phylogenetic clusters?

Much of what is known about speciation comes from the study of plants and animals [2,3,4]. For sexually reproducing organisms, recombination is tied to reproduction, and genetic homogenization of species occurs via random mating. When individuals from the same species randomly mate, and alleles at different loci evolve independently (*i.e*., are randomly associated), genetic diversity is thoroughly mixed. Therefore, arbitrary mating prevents the accumulation of independent diversity and the formation of new lineages. Should biases in mating occur (for instance in populations that maintain large habitation zones where individuals on the periphery mate infrequently with those on the opposite side), genetic differences would then accumulate separately to form diverged populations within the species. If a geographic effect persists for extended periods of time, speciation might occur. For sexually reproducing organisms, geographic isolation (allopatric speciation) is overwhelmingly the most common mechanism for speciation [7]. Since allopatric speciation is based on physical barriers to random gene flow, natural selection is not required to induce the accumulation of independent diversity and is therefore considered the null hypothesis for the generation of species. Some species have highly limited ranges of habitation and, therefore, geographic isolation does not occur. Yet, speciation (sympatric) can still proceed when random mating is disrupted by genetic characteristics [8,9]. For instance, mate choice can have a strong impact on biasing mating and sympatric speciation [10]. Unlike allopatry, the mechanisms for sympatric speciation require natural selection to act on traits that generate mating biases [9,11].

Speciation in asexually reproducing organisms is much more complicated. In fact, it is thought that species may not exist in organisms for which sexual reproduction does not occur [3]. However, absolute asexuality is rare with perhaps only bdelloid rotifers being the exclusive example of long-term clonality [12], signifying that any evidence for species in asexual organisms suggests also the presence of at least occasional sex. Interestingly, asexuality in eukaryotes seems to continually evolve from sexual ancestors, and genomic analyses are pointing towards the last universal ancestor of all eukaryotes having the capability for sexual reproduction [13]. Therefore, when looking for the evolutionary origins of sexuality, prokaryotes are likely to yield unexpected results.

It seems somewhat paradoxical that two of three domains of life reproduce asexually, and yet there exists abundant evidence for discontinuity in the distribution of genotypic and phenotypic traits in those groups [14,15,16,17,18,19,20,21,22,23,24,25,26,27,28]. It was proposed long ago that asexual populations evolve through population-wide fixations of genomes [29,30]. In the absence of gene flow, genes on a chromosome are permanently linked and share the same fate. For instance, any advantageous mutation that occurs at one locus would rise in frequency within the population due to natural selection. Because all loci are linked on the same genome, their frequency in the population would rise at the same rate. Theoretically, a genome with a single advantageous trait would outcompete the other genomes in the population until all individuals without the mutation were extinct and all survivors would have an identical chromosome [29,30]. These events are called clonal sweeps, or periodic selection events, and have been proposed to be the evolutionary homogenizing force preventing the accumulation of diversity in an asexually reproducing species. Clonal sweeps in bacteria were reported by Atwood and colleagues [31] when they noticed genetic replacement of mutants in *Escherichia coli* cultures. Further, *E. coli* population genetics studies using enzyme electrophoresis demonstrated very low rates of recombination amongst strains [32], which supported the hypothesis for clonal sweeps as the dominant evolutionary force homogenizing and maintaining prokaryote species [33]. It had been proposed that adaptation to an environment in which no direct competition between individuals from the ancestral population occurred prevented clonal sweeps and thus allowed the accumulation of independent mutations and the emergence of clustered diversity (*i.e*., species), each experiencing localized clonal sweeps [14]. However, little or no evidence for clonal sweeps has been discovered [34,35,36,37]. Rather, extensive data support alternative hypotheses for the origin and maintenance of phylogenetic clusters in prokaryotes, e.g., [19,21,25,34,35,36,37,38,39,40].

Though prokaryotes reproduce asexually, they can and do acquire DNA from other sources and use it to undergo genetic recombination. In fact, the discovery of DNA as the genetic material of all life might have taken much longer to determine. Frederick Griffith, later followed by Avery and colleagues in their now classic experiment, observed the effects of gene transfer, now called natural transformation, when they injected into mice a mixture of a live non-virulent *Streptococcus* with the components of heat-killed virulent *Streptococcus* [41,42]. Though the results seemed clear, there was some lingering doubt: DNA seemed too simple of a molecule for the complexities of inheritance. Another experiment using radiolabeled bacteriophages removed any remaining reservations regarding the role of DNA [43] and also discovered the transfer of DNA from a phage into a host, what is now referred to as transduction. While these experiments were instrumental in demonstrating the role of DNA for coding inheritance, for the purposes here, they were also investigations showing the discovery of important modes (natural transformation and transduction) for acquiring genetic information in prokaryotes, what we call now horizontal gene transfer (HGT). The role of gene transfer in the evolution of bacteria and archaea has taken a long time to uncover and has sometimes been controversial [44,45], but there seems to be little doubt as to the importance of frequently moving genes between cells. Can it account, however, for the observation of species?

In sexually reproducing organisms, it is a general principle that individuals mate only within their own species, though hybridization between species certainly occurs (e.g., [46]). Therefore, gene flow is biased almost exclusively to members of the same species, with some low amount occurring between closely related species. Similar concepts have been applied to prokaryotic species. The role of biased gene flow in prokaryotes has a long history of investigation and abundant evidence from several model systems supports the concept. Approximately 50 years ago, analyses of strains of *Pneumococcus* and *Streptococcus* [47] and different *Bacillus* species [48,49] demonstrated a strong positive correlation between genetic relatedness and the frequency of gene transfer in laboratory experiments: the more distantly related two strains were, the less efficient the recombination. A connection between the frequency of genetic exchange and speciation in bacteria was quickly made [50]. Later, the relationship between gene exchange frequency and genetic divergence was shown to be log-linear, where small changes in relatedness resulted in enormous drop-offs in homologous recombination (HR: Typically, HR in prokaryotes means the replacement of a homologous locus in a non-reciprocal process, but the actual process may be more complicated. For instance, a cell could gain, via non-homologous processes, an extra gene copy, maintain both copies for an extended period of time, but then lose the original. In such a case, it might be considered HR, though the process was not.) For example, transfer efficiency between closely related species like *E. coli* and *Salmonella typhimurium* (now called *S. enterica*, subsp. *enterica*), incurred a loss of more than four logs compared to intraspecies transfer [51]. Heavily biased HR frequency has broad implications for genetically isolating species, and was the basis for a biological species concept developed from the analysis of *E. coli* more than two decades ago [17]. Before moving on, it is important to note that horizontal gene transfer (HGT: HGT often refers the exchange of any gene between species by any process, but it can also refer specifically to HR-independent mechanism exchanges like those induced by phages, or transposons.) between species occurs, commonly traversing very large genetic distances, and inter-phylum and inter-domain transfer is well documented. For instance, a large fraction of core genes (*i.e*., genes common to all members of a group, typically highly conserved genes for replication, transcription and translation) from the deep branching bacterial order *Thermotogales* originated from archaeal and clostridial sources: horizontally transferred core genes outnumber the genes considered to have evolved from a common ancestor with the *Aquificales* [52]. Recently, it was suggested that HGT events from bacteria into archaea might be responsible for the origin of at least 13 archaeal orders [53]. The transfer efficiency across great genetic distances can be below our ability to detect them in laboratory transformation experiments, but over long time periods, the effect is real and causes dramatic changes in evolutionary trajectories, especially in comparison to the effects of mutation alone.

Besides demonstrating bias in gene transfer, it was also discovered that the frequency of recombination was high enough within many species to unlink genes on a chromosome *i.e.*, random allelic associations at different loci, or linkage equilibriums (e.g., [18,54,55,56,57,58,59,60]. The detection of high gene flow within many different populations signifies that recombination is a widespread homogenizing force preventing genetic divergence in bacteria and archaea. Further, it indicates that barriers to gene flow are likely required to promote speciation. Therefore recombination can be both a diversifying and homogenizing force, depending on the source of DNA (e.g., within *vs.* between species). To estimate the amount of recombination required to produce a random association of alleles at different loci in natural populations, the relative rates of recombination to mutation (r/m) were compared. Perhaps unexpectedly, linkage equilibrium in prokaryotes is often achieved with an r/m estimated to be around one. In the free-living marine cyanobacterium *Microcoleus chthonoplastes*, populations were measured to have random allelic associations [56], and in a separate study were estimated to have an r/m ratio of 0.8 [61]. Similar observations are made in *Sulfolobus islandicus*, where populations are considered to be in linkage equilibrium [60] and to have an r/m ratio of 1.2 [61]. The values measured in nature are very close to those determined in computer simulations of bacterial population evolution: r/m values in which recombination is a homogenizing force were determined to be as low as 0.25, and as high as 4.0 [19]. It is frequently considered that recombination in prokaryotes is too low to break up gene linkage in comparison to eukaryotes (e.g., [14]); however, the estimated r/m for many animals (including humans [62], and *Caenorhabditis* [63]), and plants (including *Arabidopsis* [64], *Brassica* [65] and pines [66]) is below one, indicating that the relative r/m rates in many prokaryotes is largely equivalent to, or higher than their sexually reproducing counterparts.

As with sexually reproducing randomly mating eukaryotes, the observation of random allelic associations in prokaryotic species means that individual loci are not linked and evolve independently. This lack of linkage combined with natural selection can cause an advantageous allele to rise in frequency at a locus yet have little or no effect on the variation at other loci [67], depending on the size of the recombining fragments [68]. Sweeps of individual genes in *E. coli*, a species not known for rampant HGT, were first reported over 30 years ago [69]. Recent population studies using advanced high-throughput sequencing techniques of DNA and proteins (e.g., genomics, metagenomics, proteomics and metaproteomics) provide additional examples of independent gene fixation events in marine organisms [37,70] and extremophiles [15]. For instance, comparative genomic analysis of 20 marine *Vibrio* strains from two recently diverged natural populations demonstrated that individual loci were being swept to fixation on a constant basis due to the combined effects of recombination and selection [37]: no evidence for genome wide fixation of genes in the population was observed. In Shapiro *et al.* [37], it was reported that in the time since the initial divergence of the two *Vibrio* populations neutral recombination was so frequent that any evidence for a clonal signature had been obscured, and that no single bifurcating tree exemplified the evolution of more than 1% of the core genes. It was suggested that adaptation to different niche spaces inhibited unbridled recombination and allowed the insipient lineages to diverge, which makes prokaryotic speciation more like eukaryotes than previously envisioned.

## 2. The Haloarchaea

Halophilic archaea, officially called Halobacteria, comprise an entire class within the domain Archaea [71,72]. Quite frequently, the synonym haloarchaea is used though the term has no taxonomic standing [73]. They are primarily characterized by their obligate requirement for high concentrations of NaCl. Metabolically, they are mostly aerobic heterotrophs [73] that thrive in moderate (15%–20% NaCl) to saturated brines (~35% NaCl), which are often anaerobic. They can experience a wide range of temperatures in a single location, but are known to thrive in the environment above 45 °C [74], and below zero (e.g., Deep Lake, Antarctica [75]). Further, many live in neutral or alkaline waters as well [76]. Therefore, this group encounters a wide variety of environmental conditions to which they must be adapted. Many haloarchaea utilize light energy to pump protons across their membrane via a rhodopsin/retinal system, which allows them to generate ATP [77,78]. However, none have been implicated in the ability to reduce carbon dioxide. The haloarchaea are typically the dominant group in environments containing greater than 15%–20% NaCl, in some cases comprising 90% of the total number of cells [79] and often encompassing a vast majority of sequencing reads in metagenomic studies [80,81,82,83,84]. Because haloarchaea use high intracellular salt concentrations (KCl), rather than producing energetically costly organic compatible solutes like ectoine or glycerol to equalize osmotic pressure [85], they likely gain a metabolic advantage in this environment over bacterial and eukaryal competitors. It is a rare circumstance that a single taxonomic order is restricted to a specific environmental condition (e.g., extremely high NaCl concentrations), and is also the dominant group in that habitat. In some sense, this unique condition provides an interesting opportunity to investigate the process of speciation. Further contributing to the species investigatory cause is the highly diverse abiotic conditions of brine pools and their widespread but patchy distribution, which provide a Galapagos Islands-like system for microbial evolutionary research.

## 3. HGT and Haloarchaeal Evolution

The haloarchaea as a group have an established reputation for undergoing a lot of HGT and HR [57,67,71,72,75,80,86,87,88,89,90]. Because the haloarchaeon *Halobacterium* sp. NRC-1 was amongst the first to have its genome sequenced it was identified early on that HGT was rampant in this group [91]. In the genome study by Ng *et al.* (2000) it was observed that a ‘substantial’ number of genes acquired by *Halobacterium* were from the Domain Bacteria, most notably from the radiation resistant species *Deinococcus radiodurans* and the Gram-positive genus *Bacillus*. Interestingly, among the identified transfers thought to be from bacterial sources was a cohort of genes necessary for aerobic respiration. A more recent study confirmed those observations of ‘substantial’ HGT from bacterial sources, and further indicated those gene transfer events were not exclusive to *Halobacterium* sp. NRC-1 but were widespread, and perhaps more interestingly occurred before the haloarchaeal last common ancestor [71]. Phylogeny of many conserved genes indicates that the ancestor of all haloarchaea was a methanogen [71,92,93] and the origin of the order Halobacteria is now hypothesized to have been induced by transfer events from bacterial sources that changed an autotrophic anaerobe into a heterotrophic aerobe [71]. Since its ancient origins, horizontal gene transfer seems to have been a major evolutionary process for haloarchaea.

Gene transfer across large genetic distances is not exclusively a process of non-homologous acquisition as might be expected from transformation experiments between species [19,51,94,95,96]. More than a decade ago, it was demonstrated that haloarchaea are quite capable of recombining homologous fragments of rRNA genes that originated from distantly related haloarchaeal genera [97], a process considered unlikely as radical changes to gene sequence in any aspect of the ribosome should ordinarily cause the death of a cell that experiences them [98,99,100]. The observation that such events occurred in haloarchaea suggests that homologous replacement happens all the time but that only a few events provide benefit and survive the selection gauntlet. Studies on halobacterial species that preserve highly divergent rRNA operons (e.g., 6% sequence difference) indicate that they are expressed under different environmental conditions [101], which offers an explanation for their maintenance in the same cell, and why gene conversion has not homogenized the divergent copies. Similar selective forces may have been key in retaining newly acquired divergent homologous rRNA replacement fragments observed by Boucher *et al*. (2004).

Evidence from a recent study on haloarchaeal genomes indicates that intergenus homologous recombination happens frequently at most loci [102]. Using a concatenated ribosomal protein gene phylogeny as a proxy for estimating the evolutionary history of the haloarchaea, Williams and colleagues showed that gene families common to all haloarchaea were recombined across great genetic distances [102]. In agreement with a selection hypothesis against highly divergent DNA while also generating “hopeful-monsters” [103], the frequency of gene exchange among haloarchaea was demonstrated to have a log-linear relationship with genetic relatedness, but that the slope was not steep and there were no absolute barriers to homologous recombination. Every haloarchaeal genome examined was capable of HR with any other haloarchaeon irrespective of relatedness, only the probability of it occurring changed. Living together in similar habitats, and at high cell density may contribute to frequent gene exchange, but homologous replacement between very distantly related organisms is unlikely to be restricted to the haloarchaea. Two other important observations were made by Williams *et al.* [102]: (i) genes originating from even distant relatives were almost certainly replaced through HR processes, rather than through the acquisition of a second copy followed by the loss of the first; and (ii) that single genes, or fractions of them were being replaced, as apposed to multiple adjacent genes, or operons. Indeed, single gene replacements within operons were evident. That single genes were observed as being replaced seems slightly mysterious because the only mechanism demonstrated for gene transfer in haloarchaea has shown that enormous DNA fragments (>500 kb) are recombined [86]. Similar large recombination fragments were observed in natural populations from Antarctica: DNA as large as 35 kb were observed to be recombined across genera [75]. Therefore, it seems enigmatic that evidence for large multi-gene DNA fragments are lost when a wide range of haloarchaeal diversity is examined. Which evolutionary processes obscure large fragment exchange events? Maybe mating more frequently recombines gene-sized fragments as the Naor *et al*. [86] experimental conditions required large fragments to be recombined, and any small fragments went undetected? Perhaps mating is not the dominant HGT mechanism, with viruses and conceivably natural transformation transferring smaller DNA fragments and playing a much larger role? Certainly, more investigation into the mechanisms of mating, transduction and natural transformation are needed to answer these questions.

The frequency of homologous replacement of loci between species appears to be higher in the haloarchaea than in other tested bacterial model organisms. A laboratory study that measured directly the frequency of recombination between *Haloferax volcanii* and *Haloferax mediterranei* that exhibit ~14% nucleotide sequence divergence across shared orthologous genes demonstrated a drop in efficiency less than two orders of magnitude compared to intraspecies measurements [86]. In contrast, recombination between *E. coli* and *S. typhimurium* (*S. enterica* subsp. *enterica*), and between different species of *Bacillus*, which are similarly divergent, showed about four orders of magnitude difference between, compared to within species [51,96]. However, there are limited numbers of studies that estimate the frequency of recombination directly using model organisms, most of which have been detailed above. With the low cost of DNA sequencing, estimates for recombination are not measured directly but obtained from populations and derived from the relative r/m rates. Sequence data from population genetic studies suggests that haloarchaea experience modest rates of recombination compared to many others [61]. Now that many more genetic systems are available for directly measuring recombination *versus* genetic distance, haloarchaea may eventually lose their status of highest gene replacement rates for a model organism.

Population genetics analysis on closely related strains (<1% nucleotide sequence divergence for five core genes) that formed tight phylogenetic clusters called phylogroups indicated that many species of the genus *Halorubrum* are highly recombinogenic. Using multilocus sequence analysis (MLSA) featuring the PCR amplification and sequencing of five conserved loci (16S rRNA, *atpB*, bacteriorhodopsin, *EF*-2 and *radA*) from hundreds of strains revealed several important observations regarding the evolution of the haloarchaeal genus. Of notable consequence was the observation that three different *Halorubrum* populations, probably representing three unique species depending on which sequence cutoffs were applied [67], were undergoing HR frequently enough that each of them was in genetic equilibrium [57,88]. Genetic equilibrium as a reminder occurs when all alleles at the observed loci are randomly associated, which is the expectation for sexually reproducing randomly mating species. To estimate the r/m required to attain a random association amongst alleles, single locus variant analysis was employed [104,105]. This analysis determined that for every mutation detected, recombination changed eight nucleotides [88]. Further evidence supporting a strong HR homogenizing effect was the observation that the same bacteriorhodopsin allele was found in all strains of two *Halorubrum* spp. phylogroups, while the other four loci examined had high diversity [88]. Furthermore, because each studied locus had differing amounts of variation, each one must have been fixed in their respective populations at different times. This indicates that advantageous genes are being obtained constantly, either through mutation or gene transfer from other species, and being continually independently fixed in the population. Therefore, the loci on all the chromosomes within each population are unlinked by recombination and fixed by natural selection (and possibly genetic drift; [106]) one locus at a time.

New alleles in a population can originate within the population by mutation, or they can be acquired by HGT. If mutations are the dominant source of fixed variation, then the expectation is that most genes would have a similar phylogenetic signal both within and between species. However, if HGT is the dominant source of fixed variation, then all individuals inside the population would be related to each other, and different loci would have alternative evolutionary relationships between species. In the case of *Halorubrum* isolates detailed above, the phylogeny of each gene reconstructed the same phylogroups. However, each gene phylogeny showed a different relationship between the phylogroups, and all possible relationships were robustly recovered. Furthermore, some genes had strongly supported multiple phylogenetic signals, indicating smaller intragenic DNA fragments were transferred [67,88]. Until genomes are analyzed, it is difficult to know the extent of interspecies gene transfer within *Halorubrum* populations, however it does appear from MLSA that a significant fraction of core gene diversity is derived from acquiring other species’ genes, and then fixed in the population.

Chromosome dynamics amongst the haloarchaea due to HGT, genome rearrangement, and gene loss is a powerful evolutionary force. Evidence for this was first witnessed from the analysis of metagenomic sequence data obtained from a saltern crystallizer pond (e.g., saturated brine with precipitated NaCl) in Santa Pola, Spain that is comprised almost exclusively of *Haloquadratum* sp. cells [79,81,82]. This data was compared to the sequenced genome of *Haloquadratum walsbyi* strain HB001 cultivated from the same pond [80,107]. Analyses showed that the isolated strain, which was derived from a single environmental cell, had multiple regions in its chromosome that were not represented elsewhere in the environmental *Haloquadratum* population DNA (*i.e*., were unique to that strain, called genomic islands) [80,83]. Finding these genomic islands profoundly suggests that each cell in the environment might contain a distinct genome [108]. In a limited test of that hypothesis for haloarchaea, a recent study reported the diversity of *Halorubrum* and *Haloarcula* strains cultivated at the same time on the same media using water from the same few microliters of hypersaline lake brine [89]. The study showed that strains sharing >99% sequence identity for five core genes also had unique genomes. Whole genomes were fingerprinted by implementing primers that annealed randomly to the chromosome, which amplified arbitrary fragment numbers and sizes. Gel electrophoresis revealed banding patterns generated by the amplification process, and thus provided a genomic fingerprint. Remarkably, even strains with identical haplotype sequences had different genomic fingerprints [89]. Genome sequencing of a selected subset of those strains confirmed that each genome was distinct by revealing that each one had a different size, even those that had identical core gene sequence data were different by up to 500 kbp [109]. These analyses using different methodologies and genera suggest haloarchaeal genome flux is faster than the rate of neutral mutation, and speculatively as frequent as every generation.

The above observations for haloarchaea support an evolutionary scenario of constant and high inter- and intra-species recombination that breaks linkage of loci in populations. Selectively advantageous newly transferred alleles rise in frequency in the population until all cells have the same copy, suggesting fixation occurs faster than the neutral mutation rate can cause a mutation in the new allele. Successful HR events are also more likely if the DNA originated from closer relatives, with intra-species gene exchange the most efficient. Once fixed, neutral mutations begin to accrue providing diversity at the locus. Most loci get an advantageous allele from HR rather than from mutations within the species. Further, the species gene content variation (*i.e*., pan-genome) is enormous with the distinct possibility that every cell in a population is unique. This variation may possibly be acquired every generation by gene transfer and loss. Despite clonal reproduction, evidence for two strains being identical is absent from the data. The observed maintenance of phylogenetic lineages in the face of extensive interspecies and intergenera recombination is more than likely determined by who the frequent trading partners are—intra-species occurs more frequently than inter-species, which is more frequent than intergenus and interfamily transfer [110].

## 4. Haloarchaeal Speciation

It is fair to avoid a long discussion of species, other than to say taxonomically speaking species descriptions for the Halobacteria are based on the analysis of a type strain, which is deposited into a culture collection and to which all subsequent strains are compared when trying to decide if a strain belongs to a previously described or undescribed species [73,111]. Any strain with greater than 3% 16S rRNA gene sequence divergence from any type strain is considered a new species [112]. For those strains with less than 3% divergence, if they have less than 70% DNA-DNA hybridization values compared to any type strain, they are considered a new species. This is a technical and pragmatic solution to a complicated problem, and, therefore, does not consider the diversity of individuals in a species nor the evolutionary forces that sculpted the divergence. For the permanent accrual of independent variation in highly recombinogenic populations such that two new species are recognized by the taxonomic code, gene exchange trading partners must become biased, either sympatrically or allopatrically. How might this occur?

Sympatric speciation for highly recombinogenic bacterial populations has been modeled on adapting to a new niche [25,39,113,114]. Lawrence (2002) argues persuasively and supports his ideas with data [113,114,115] that sympatric speciation can occur. His model requires that a cell obtains a gene or operon via HGT that is required for survival in a new niche, and therefore if lost, will result in the death of the cell. Because the cost of losing the gene is high, this localized region of the chromosome cannot be homogenized by recombination with cells in the population that do not have the gene, and therefore neutral mutations begin to accrue in the flanking regions. Meanwhile, HR and homogenization continues with close relatives throughout the rest of the chromosome. As HR in that localized region declines and neutral mutations increase, a ratchet effect occurs that extends the size of the non-recombining region. As time progresses, it is feasible that mutations will eventually accrue to the point that they alone can drive speciation. However, if additional HGT events occur and seed further regions of limited recombination, speciation is expedited. Recent analyses by Friedman and colleagues using computer simulations to assess sympatric speciation found echoes of Lawrence’s concepts in that they show an effective solution for permanent divergence can be initiated by the acquisition of a small number of niche adaptive genes that promote ecological differentiation [39]. While adaptive genes might promote sympatric speciation in haloarchaea, there is no study demonstrating evidence for such a process. Data does exist showing that closely related species co-occur and have apparent ecological differences, e.g., [75,116], but this is not evidence of speciation in the same place.

The null hypothesis of geographic isolation providing barriers to recombination may prove fruitful as a mechanism for generating species. Hypersaline environments are scattered across the earth’s surface, and on sea floors. This irregular distribution means that for a species to exist genetically thoroughly mixed everywhere (*i.e*., panmixis), dispersal to all locations must be fast and constant. If, on the other hand, the accumulation of genetic differences (e.g., localized HGT and mutations) is faster than the rate of dispersal, then location-specific variation will accrue and allopatric speciation could occur. There are no studies testing either of these conditions directly, however several studies are beginning to show evidence favoring a biogeographic effect.

First, it is important to point out similar genera and species are found widely distributed, perhaps carried by seabirds [117]. For instance, the genus *Halorubrum* is frequently reported in different hypersaline environments located in geographically distributed locations e.g., [81,88,118,119,120,121,122,123], and the species *Haloquadratum walsbyi* is described from two strains cultivated from Australia and Spain [107,124,125,126]. Indeed, a wide distribution for *Haloquadratum walsbyi* is reported [123]. These data indicate that dispersal occurs, that it likely happens at a high rate, and it may prevent geographic isolation.

However, there are several lines of evidence that not everything is everywhere, and that the rate of evolution may be faster than the rate of dispersal. Beginning with community composition, it has been shown that studies comparing geographically separated hypersaline environments are quite dissimilarly composed. A metagenome study comparing two different solar salterns located on different coasts of Spain (one on the Mediterranean, the other on the Atlantic) reported that communities were very different from each other, likely the result of ecological conditions at each site [81]. Analysis of community structure using cell sorting based on DNA stain fluorescence and light scatter and 16S rRNA gene sequencing showed that haloarchaeal communities from three different saturated saltern ponds located in Spain, Tunisia and California had statistically differently community structures, and that sequences found in California and Tunisia were mostly unique to their locations [127]. Further, the same study showed that the California site was unexpectedly composed of a wide diversity of bacteria, and that bacteria comprised approximately 50% of the analyzed community. Another study from a solar saltern in Baja California, Mexico showed that saturated brine pond communities were different from those in Spain, and like the community in California from the Zhaxybayeva *et al*. (2013) study, contained an unexpectedly wide diversity of bacteria [128]. These studies indicate that communities are differentially composed, and that conditions exist that prohibit the dispersal and invasion of certain species at every location. Remarkably, all of the studies detailed above are from brines originating from seawater, meaning the salt concentrations and ratios are highly alike. If comparably structured hypersaline environments were to exist, seawater derived solar salterns would have the highest probability of having such a community. Natural lakes are typically composed of salts carried from the surrounding geological strata and can vary significantly from lake to lake, therefore they are not expected to have similar community structure. Similar arguments could also be made for salt springs and deep-sea brine pools too. Therefore, we predict that while hypersaline environment will have some species and genus overlaps between some of them, they are each uniquely composed. Analyses which may shed light on this subject would be to examine metagenomic data for identical sequences in different locations, or to construct phylogenetic trees of metagenomic data and search for clusters containing sequences located from one location or another.

The genus *Halorubrum* is the largest of the haloarchaea, currently represented by 28 species [119] and is typically a dominant or co-dominant member of hypersaline environments with the genus *Haloquadratum* [81,129]. Examination of *Halorubrum* strains that appear to be representatives of the same species but which were cultivated from different locations show patterns of geographic differences. For instance, analysis of ~150 *Halorubrum* strains cultivated from Spain and Algeria, and which were greater than 99% identical for five MLSA loci demonstrated allelic and haplotype distribution patterns consistent with geography: the vast majority of alleles and haplotypes were unique to the site of cultivation [88]. Out of all the strains sequenced for five genes, only one haplotype was found common to each site, indicating that dispersal was certainly happening but not frequently enough to prevent site-specific accumulation of diversity. A recent study that analyzed the genomes of *Halorubrum* isolates cultivated from the Aran-Bidgol Lake in Iran, and *Halorubrum* genomes in the public databases also revealed evidence for a geographic effect. Phylogenetic clusters that had greater than 99% MLSA DNA sequence identity and greater than 96% average nucleotide identity (ANI) across all shared orthologs were only coherent groups when using additional analyses if they were cultivated from the same location [109]: all the strains cultivated from Aran-Bidgol that formed species like phylogenetic clusters also had similar tetramer frequencies and G+C content. One cluster, phylogroup D, exhibited statistically relevant differences in tetramer frequency and G+C content, and was composed of strains originating from different geographic locations. Of high interest was that Phylogroup D, while clearly exhibiting species-like characteristics in that they had >99% MLSA and >96% ANI similarity (which conformed to species cutoffs as being the same species [24,130]), was actually comprised of four different named species, meaning there were enough differences amongst those strains, including the gold standard of taxonomy, <70% DNA-DNA hybridization, to be differentiated taxonomically. Whether or not they are the same species is not at issue, only that there are statistically relevant differences between the strains, and that those differences were not observed when strains of similar sequence diversity were cultivated from the same location. Since they were cultivated from different locations, the simplest explanation for the accumulation of independent variation is that they were recently geographically isolated. Along similar lines, the species *Halorubrum chaoviator* represented by three strains cultivated from three locations in Greece, Australia and Mexico, all show clear differences in their polar lipid content [122], suggesting geographic isolation is driving phenotypic variation in an otherwise genotypically coherent species. Extensive analysis of *Halorubrum* genomes, and polar lipids, from strains that form tight phylogenetic clusters and cultivated from many different locations could impart additional insight into how diversity is distributed and whether or not geographic patterns are robust.

*Haloquadratum* is also a widely distributed dominant or co-dominant genus in hypersaline environments [79,123,129,131]. *Haloquadratum* seems to have restricted diversity in comparison to *Halorubrum*, containing only one species, *Haloquadratum walsbyi* [126]. Genome analysis of the two cultivated strains that represent *Haloquadratum walsbyi* (one from Spain and the other from Australia) showed limited 16S rDNA (99.9%) as well as genome-wide orthologous gene sequence diversity (98.6%), and that the genomes were largely syntenic differing primarily by gene gain and loss [132]. These observations led the authors to conclude *Haloquadratum walsbyi* is efficiently dispersed globally. However, genome assemblies derived from metagenomic data collected at Lake Tyrrell, Australia indicate that the local Lake Tyrrell *Haloquadratum* genomes are more closely related to themselves than they are to the cultivated *Haloquadratum walsbyi* strains [116]. This suggests the possibility that genotypic variation can accumulate independently in different sites separated by only a couple hundred miles (e.g., distance from Lake Tyrrell to Cheetham Salt Works where the Australian *Haloquadratum walsbyi* was cultivated). Though 1.4% DNA sequence divergence across shared orthologous genes for each of the cultivated strain’s genomes is not very much, to us, it suggests those two strains could belong to different species. For instance, analysis of hundreds of *Halorubrum* isolates using MLSA demonstrated they always formed phylogenetic clusters with less than 1% sequence divergence [88,89,133]. Further, analysis of lipid composition, which is a typical conserved marker important for taxonomy, showed that one of the two *Haloquadratum walsbyi* strains did not contain phosphatidylglycerol [126]. While it is reasonable to consider the above details as evidence for a considerable amount of intraspecies *Haloquadratum* *walsbyi* diversity, *Halorubrum* strains in comparison tend to be more similar to each other when they come from the same location, than strains cultivated from different sites [88,109,122] suggesting *Haloquadratum* diversity might also reflect a biogeographic signature. Because *Haloquadratum walsbyi* is difficult to cultivate, reliance on metagenomic data for analyses between sites is likely the only solution to testing geographic hypotheses.

Though *Salinibacter ruber* is not a haloarchaeon, it can provide insight into spatial distributions since it co-exists with haloarchaea, and it is often a dominant bacterial species in the hypersaline environment [79]. A study detailing the distribution of genetic and phenotypic diversity of *Salinibacter ruber* isolates from around the Mediterranean Sea, the Canary Islands and Peru indicated that it was difficult to detect phylogenetic patterns of geography, but a more sensitive technique analyzing strain metabolites (metabolomics) showed clear data displaying geographic patterns, and not just for the most distant places: even sites from the Mediterranean Sea were differentiated [134]. The accumulation of independent variation within strains of *S. ruber* by site is consistent with limitations to dispersal and allopatric speciation.

Temporal studies are suggesting that haloarchaeal communities are highly stable, changing only in abundances of species according primarily to ionic concentrations, and, therefore, are resistant to invasion by dispersed cells. In a study from Lake Tyrrell, Australia, seasonal sampling and deep sequencing of community DNA revealed that *Haloquadratum* and *Halorubrum* were co-dominant genera, and negatively correlated with each others abundances, which were correlated to Mg^2+^ concentrations [129]. Similar results were obtained from the Sfax solar saltern in Tunisia where analysis showed a highly stable community structure through the seasons, and years, and that ion concentrations and temperature could explain 95% of the observed changes [135]. Stable microbial communities are resistant to invasion [136,137] and the observations of established haloarchaeal communities changing only in relative abundance suggest that even if dispersal should occur, invading might be very difficult. Imagine that one million cells from the same species recently dispersed to a new location that was filled with an established community, in which every niche is filled. To found a new population high enough to be detected, those million cells would need to out compete a vast proportion of the cells already existing (assume 10^7^/mL density, and 1000 L) and, presumably, optimally adapted to those conditions. Invasion inevitably happens, but the odds are stacked against it. These data support the hypothesis that localized evolution is faster at creating divergence between geographic populations than dispersal and invasion is at homogenizing them.

It goes almost without saying that more work needs to be done on populations representing closely related strains cultivated from different locations, and metagenomic sequence data obtained from around the globe in order to obtain a more robust vision of how diversity is distributed. That said, from the data in hand, it is possible to see evidence of geographic patterning, and this suggests that the rate of dispersal is slower than the rate of evolution for haloarchaea. Geographic barriers to gene flow therefore probably represent the simplest explanation (*i.e*., the null hypothesis) for how divergence is initiated and speciation might proceed. Once a small amount of divergence has accumulated, the data indicate that maintaining a genetically separated status is fairly straightforward, despite the possibility for populations to recolonize the same location, as cells recombine more frequently with more similar genotypes.
